# Percutaneous Transhepatic Embolization of a Bleeding Colic Vein in a Cirrhotic Patient With Massive Hematochezia: A Case Report and Literature Review

**DOI:** 10.7759/cureus.25736

**Published:** 2022-06-07

**Authors:** Aaroh Parikh, David Leon, Mohammad Ghasemi Rad, David Wynne, Amar Amaresh

**Affiliations:** 1 Department of Radiology, Baylor College of Medicine, Houston, USA

**Keywords:** ir-guided embolization, transvenous embolization, colonic varices, ectopic varices, variceal hemorrhage

## Abstract

Ectopic varices are an uncommon cause of gastrointestinal bleeding in patients with portal hypertension. A 43-year-old female with alcoholic cirrhosis developed massive hematochezia and hemorrhagic shock, requiring emergent angiography and image-guided intervention. Angiography revealed active extravasation from a branch of the right colic vein. The patient underwent percutaneous transhepatic embolization of the bleeding colic vein with technical success demonstrated on post-embolization angiography. Treatment of bleeding ectopic varices may require endoscopic, image-guided, or surgical approaches.

## Introduction

Gastrointestinal bleeding is a frequent and potentially life-threatening complication in patients with decompensated liver cirrhosis. Whereas the vast majority of gastrointestinal bleeding in cirrhotic patients arises from esophageal and gastric varices, other so-called “ectopic” varices are responsible in a small minority of cases [1]. Image-guided interventions to treat variceal bleeding range from portosystemic shunt creation to variceal embolization/obliteration. In this report, we present the case of a patient with alcoholic cirrhosis who developed massive hematochezia and hemorrhagic shock from a branch of the right colic vein that required percutaneous transhepatic embolization.

## Case presentation

The patient was a 43-year-old female with a reported history of alcohol use and cirrhosis who was admitted to the medical intensive care unit for severe hyponatremia and decompensated cirrhosis with hepatic encephalopathy. Alcoholic cirrhosis was considered the most likely etiology of her liver disease, as workup for alternative causes was negative. Labs on admission were remarkable for elevated liver function tests, including total bilirubin of 19.4 mg/dL that increased to a peak of 31.7 mg/dL during her hospital course. An ultrasound of the right upper quadrant was notable for cirrhotic liver morphology and hepatofugal flow of the main portal vein without additional signs of portal hypertension. Labs were also notable for normocytic anemia and coagulopathy (initial hemoglobin 5.8 g/dL and international normalized ratio (INR) 2.8) thought to be multifactorial with contributions from cirrhosis and poor nutrition.

**Figure 1:**
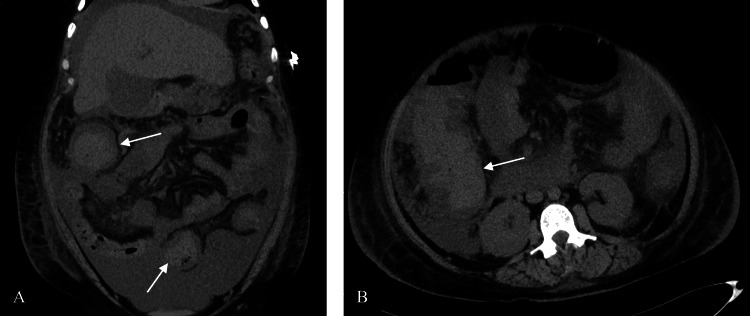
Pre-procedure computed tomography examination. Coronal (A) and axial (B) images from a computed tomography CT scan of the abdomen and pelvis without contrast reveal hyperdense intraluminal contents (arrows) throughout the colon, compatible with blood products.

Her hospital course was complicated by increasing leukocytosis and multiple episodes of watery diarrhea. A computed tomography (CT) scan of her abdomen and pelvis without contrast revealed hyperdense intraluminal contents throughout the colon, compatible with blood products (Figure 1).

Soon after the patient returned from her CT examination, she developed large-volume hematochezia resulting in hemorrhagic shock that required massive transfusion and hemodynamic support with multiple vasopressors. Her hemoglobin decreased from 8.6 to 4.0 g/dL while her INR increased from 2.7 to 5.9 in less than 24 hours. Her Model for End-Stage Liver Disease (MELD) score was 38. She underwent emergent upper and lower endoscopy, which revealed small, non-bleeding esophageal varices and large-volume hematochezia resulting in poor visualization of the colon. Interventional radiology was consulted for emergent angiography and intervention.

**Figure 2:**
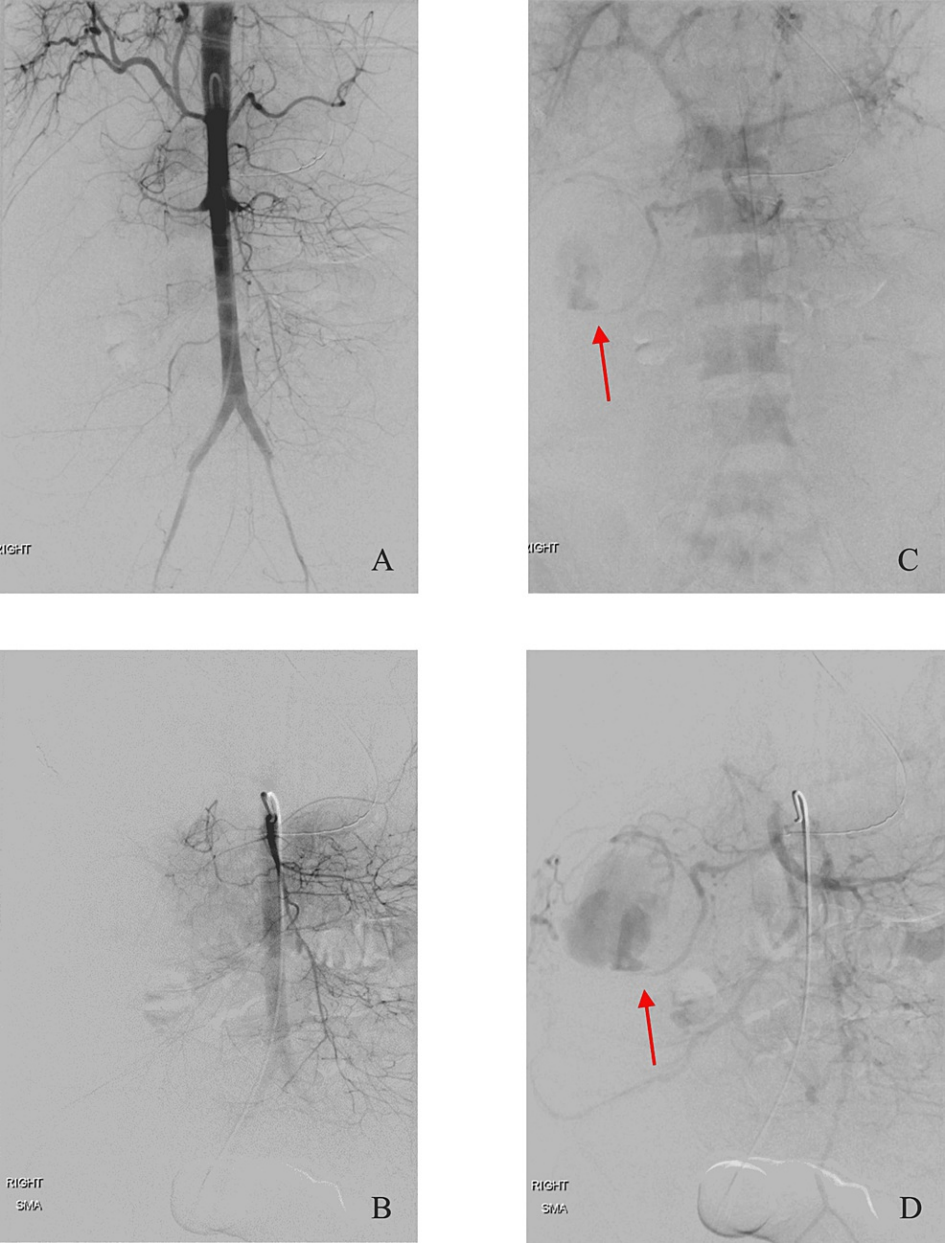
Emergent angiography. Digital subtraction angiograms of the abdominal aorta (A) and superior mesenteric artery (B) do not show any active arterial extravasation. Delayed images from the respective angiograms (C and D) reveal active extravasation (arrows) from a branch of a right colic vein into the lumen of the colon.

The patient was brought emergently to the Interventional Radiology suite. Right common femoral access was obtained, and digital subtraction angiograms of the abdominal aorta, celiac artery, and superior mesenteric artery were performed using a 5-French flush pigtail catheter for the aortogram and a 5-French SOS Omni catheter (Angiodynamics, Latham, NY, USA) for the celiac and superior mesenteric angiograms (Figure 2).

There was no active arterial extravasation. However, delayed phase images revealed active extravasation from a branch of the right colic vein on all three angiograms (Figures 2C, 2D). Embolization of the offending vessel would require portal venous access using either a percutaneous transhepatic route or the creation of a transjugular intrahepatic portosystemic shunt (TIPS). Due to the prohibitive risk of TIPS in this patient with a MELD score of 38, the decision was made to obtain percutaneous transhepatic access to the portal venous system.

**Figure 3:**
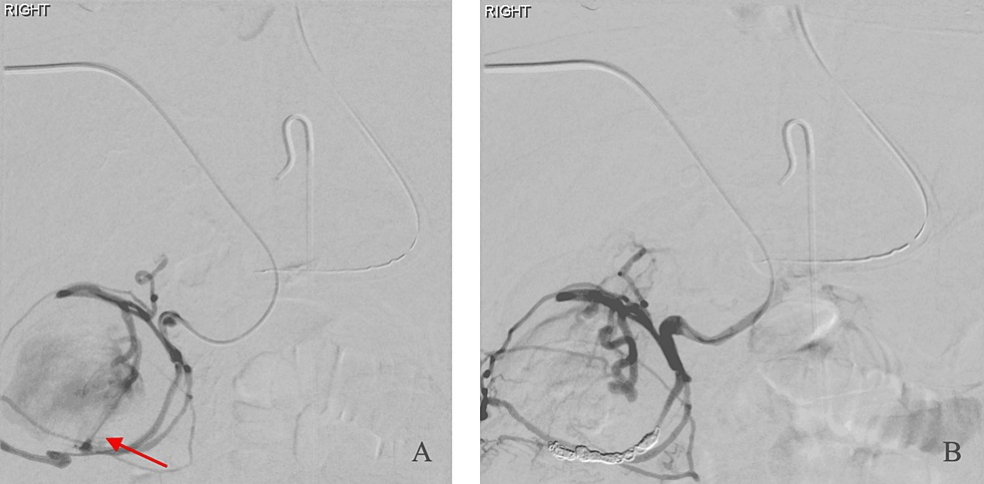
Percutaneous transhepatic embolization of the bleeding colic vein. The right colic vein was selected using a 4-French hydrophilic-coated catheter and guidewire, with a digital subtraction angiogram (A) showing active extravasation (arrow) from one of its branches. After embolization with multiple coils and gelatin sponge particles, a digital subtraction angiogram (B) shows complete stasis of flow and cessation of extravasation.

Percutaneous access into a branch of the right portal vein was achieved under ultrasound guidance using a 21-gauge Chiba needle, a 0.018 in microwire, and an AccuStick introducer system (Boston Scientific, Marlborough, MA, USA). Subsequently, a 180 cm Rosen wire was advanced through the introducer and into the main portal vein. The introducer was exchanged for a 6-French 25 cm sheath over the wire. Digital subtraction angiogram of the portal venous system redemonstrated active extravasation from a branch of the right colic vein. Notably, no varices were seen. The right colic vein was selected using a 4-French angled catheter and guidewire, with a selective digital subtraction angiogram confirming active extravasation from one of its branches (Figure 3A). The branch was selected using a 2.4-French microcatheter (Progreat, Terumo Medical Corporation, Somerset, NJ, USA). After the size of the vessel was measured using images from the angiogram, distal-to-proximal embolization was performed using multiple detachable coils. A combination of two Tornado (Cook Medical, Bloomington, IN, USA) and four Concerto (Medtronic, Minneapolis, MN, USA) embolization coils ranging in size from 3 mm × 2 cm to 8 mm × 30 cm were deployed. The coils were not completely occlusive as there was residual flow across the coil packing on a digital subtraction angiogram. As a result, gelatin sponge particles (Gelfoam, Pharmacia and Upjohn Co., Kalamazoo, MI, USA) were deployed. Although Gelfoam is not typically used to embolize arterial sources of colonic bleeding due to the risk of bowel infarction, this risk is much lower in venous embolization. Repeat digital subtraction angiogram of the right colic vein revealed complete stasis without further extravasation (Figure 3B).

Because there was no arterial source of bleeding identified on earlier angiograms of the aorta, celiac artery, and superior mesenteric artery, post-embolization angiography of the arterial system was not repeated. Due to the size of the portal venous access site (6 French), as well as the presence of portal hypertension and coagulopathy, the decision was made to embolize the hepatic parenchymal tract. The 6-French sheath, 4-French base catheter, and microcatheter system were withdrawn under ultrasound guidance until it was visualized entirely within the hepatic parenchyma. The tract was then embolized using six Concerto coils (measuring 4 mm × 10 cm and 5 mm × 20 cm) followed by Gelfoam pledgets. Immediate post-embolization grayscale and color Doppler ultrasound images revealed small-volume ascites without bleeding at the liver capsule access site.

Following completion of the procedure, the patient returned to the medical intensive care unit. She continued to have active bleeding per rectum as well as from other orifices and line insertion sites, with laboratory studies supporting a diagnosis of disseminated intravascular coagulation (DIC). She developed multiorgan failure and subsequently expired on post-procedure day two.

## Discussion

Gastrointestinal bleeding is a common and potentially life-threatening complication of decompensated cirrhosis and portal hypertension. Etiologies include esophageal or gastric varices and, less frequently, portal hypertensive gastropathy or so-called “ectopic” varices. Ectopic varices, defined as dilated portosystemic collaterals located outside the esophagus or stomach, are far less common than gastroesophageal varices, accounting for only 1-5% of all gastrointestinal bleeding in patients with portal hypertension [1].

Because there are no randomized clinical trials that elucidate the optimal therapy for ectopic varices, treatment options are manifold and should be considered on a case-by-case basis in consultation with gastroenterology, interventional radiology, and surgery. Endoscopic therapy with sclerotherapy, cyanoacrylate injection, or band ligation can be effective treatments for ectopic varices, including colonic varices [2,3]. TIPS creation and variceal embolization have been used to treat ectopic varices both as primary intervention and as rescue therapy in cases refractory to endoscopic treatment [4,5]. Similarly, balloon-occluded retrograde transvenous obliteration can successfully treat ectopic varices [6,7]. Percutaneous transhepatic obliteration (PTO) has also been reported as a treatment option for ectopic varices [8-10]. In addition to the risk of transhepatic tract bleeding, PTO carries a risk of recurrent variceal bleeding in the absence of portal decompression [11]. Although surgery is associated with significant morbidity and mortality in patients with decompensated liver disease, dearterialization and resection of bleeding ectopic varices have been successfully reported [12,13].

There is a limited number of case reports of colonic varices successfully managed by image-guided intervention. Ko et al. report a case very similar to ours, in which a young female with alcoholic cirrhosis presented with massive hematochezia and was found to have a bleeding varix in the distal ascending colon [14]. This patient was managed with coil and glue embolization of the offending varix with subsequent cessation of hematochezia and stabilization of hemoglobin, although the patient later succumbed to multiorgan failure. Wiegand et al. report a similar case of a male with alcoholic cirrhosis who presented with massive hematochezia and was found to have a bleeding varix in the ascending colon [15]. This patient was initially managed with TIPS but developed re-bleeding on post-procedure day one and required coil and glue embolization. Lastly, Reddy et al. report a case of a male with alcoholic cirrhosis who also presented with massive hematochezia and was found to have bleeding varices in the cecum [16]. The patient underwent TIPS but very soon developed DIC and expired due to multiorgan failure.

Our patient did not have a known history of varices or gastrointestinal hemorrhage prior to admission. Her initial imaging studies showed hepatofugal flow in the main portal vein and ascites, consistent with portal hypertension. Her upper endoscopy did indeed reveal small, non-bleeding esophageal varices. Yet, her mesenteric and portal venous angiograms did not show any varices. Although ectopic varices can present as the initial manifestation of portal hypertension, isolated colonic varices are extremely uncommon [11]. It is possible that in the setting of massive ongoing bleeding and intravascular depletion, the portal system was decompressed, precluding visualization of definite varices. Rare causes of ectopic varices in the absence of portal hypertension include arteriovenous fistula, congenital portosystemic anastomosis, familial colonic varices, or thrombosis of intra-abdominal vessels [17-20]. However, the patient did not have any history or angiographic evidence to support these alternative etiologies. Although diverticulosis can cause lower gastrointestinal bleeding, the patient’s CT did not show colonic diverticula. The patient developed DIC, which commonly presents with superficial and mucosal bleeding (e.g., petechiae, ecchymoses, oozing from line insertion sites). Gastrointestinal bleeding can be a manifestation of DIC, albeit considered to be much less common. Thus, although the definite etiology of her hemorrhage remains uncertain, a bleeding ectopic varix is the most likely cause given her history of cirrhosis and coagulopathy.

## Conclusions

In this report, we present the case of a patient with alcoholic cirrhosis who developed massive hematochezia and hemorrhagic shock from a bleeding colic vein treated by percutaneous transhepatic embolization with multiple coils and gelatin sponge particles. Technical success was achieved and confirmed with complete stasis and cessation of active extravasation on post-embolization angiography. However, the patient was not a candidate for TIPS and ultimately succumbed to multiorgan failure following the procedure. Although varices were not visualized angiographically at the time of the procedure, a bleeding colonic varix is the most likely etiology given the patient’s history. Ectopic varices are an uncommon cause of gastrointestinal bleeding in patients with portal hypertension. Treatment options, including image-guided interventions, should be considered on a case-by-case basis with multispecialty consultation.
